# The epidemiology of norovirus gastroenteritis in China: disease burden and distribution of genotypes

**DOI:** 10.1007/s11684-019-0733-5

**Published:** 2019-12-10

**Authors:** Honglu Zhou, Songmei Wang, Lorenz von Seidlein, Xuanyi Wang

**Affiliations:** 1 Key Laboratory of Medical Molecular Virology of MoE & MoH, and Institutes of Biomedical Sciences, Fudan University, Shanghai, 200032 China; 2 Laboratory of Molecular Biology, Training Center of Medical Experiments, School of Basic Medical Sciences, Fudan University, Shanghai, 200032 China; 3 Mahidol-Oxford Tropical Medicine Research Unit, Faculty of Tropical Medicine, Mahidol University, Bangkok, 10400 Thailand; 4 Centre for Tropical Medicine and Global Health, Nuffield Department of Medicine, University of Oxford, Oxford, OX1 2JD UK; 5 Children’s Hospital, Fudan University, Shanghai, 200032 China

**Keywords:** molecular epidemiology, norovirus, disease burden, genotype, China

## Abstract

With the improvements of sanitation and nationwide safe water supply the occurrence of bacterial diarrhea declined remarkably, while viruses became the leading causes of acute gastroenteritis (AGE). Of these viruses, noroviruses (NoVs) are responsible for a considerable burden of gastroenteritis, especially in children < 2 years and elderly ⩾ 65 years. NoVs circulating in the Chinese population are antigenically highly diverse with the genotype GII.4 being the dominant strain followed by GII.3. Given the widespread contamination in environmental sources, and highly infectious nature of NoVs, vaccination would be the desirable strategy for the control of NoV infections. However, a better understanding of acquired immunity after infection, and a reliable immunological surrogate marker are urgently needed, since two vaccine candidates based on virus-like particles (VLPs) are currently moving into clinical evaluations in China.

## Introduction

Despite significant advances in sanitation improvement, safe water supplies, and vaccination, diarrheal disease remained worldwide the fourth most frequent cause of death for children < 5 years of age in 2016 [1]. Globally, noroviruses (NoVs) was associated with approximately one-fifth of all diarrhea cases [2]. Young children experienced the highest incidence of disease, while severe outcomes were most common among young children and the elderly [3]. Despite the extensive diversity of NoVs, a single genotype (GII.4) is most prevalent in humans worldwide [4,5]. In China, according to the National Notifiable Infectious Disease Reporting (NIDR) system, 1 275 290 cases of infectious diarrhea were reported in 2017 (excluding cholera, dysentery, and enteric fever), resulting in an incidence of 92.2/100 000 person/year [6]. Among 9.3 percent of infectious diarrhea cases with laboratory confirmed pathogens, viral infections were responsible for 91.0% [7]. NoVs play an increasingly important role in the etiology of diarrhea in China [8].

NoV is a non-enveloped, small RNA virus that contains a single stranded, positive-sense, polyadenylated RNA genome. It has an icosahedral shape with a diameter of about 38 nm. The genome is approximately 7.4–7.7 kb in length and consists of three open reading frames (ORFs). ORF1 encodes six non-structural proteins, including RNA-dependent RNA polymerase (RdRp), which plays a major role in NoV replication in host cells. ORF2 is approximately 1.8 kb in length and encodes the major structural protein, VP1, including the highly conserved inner domain (S domain) and the protruding domain (P domain). The P domain consists of a stem-like, moderately conserved P1 subdomain and a surface-exposed, hypervariable P2 subdomain, thus determining the antigenicity and cell binding of the virus [9]. ORF3 is approximately 0.6 kb in length, encoding a 22kDa small structural protein VP2, may be related to forming of a portal-like channel through the capsid for delivering viral genome into host cells [10]. NoV genotyping showed that the virus has high genetic and antigenic diversity, including five gene groups, of

*Honglu Zhou and Songmei Wang contributed equally to this work.

which GI and GII groups are mainly related to humans, with 9 and 22 genotypes, respectively [11].

NoVs are highly infective for a range of reasons. First, the minimum infectious dose is less than 10 copies of the virus [12]. Second, the infected person has a long virus shedding period [13], and even continues to be infectious after the clinical symptoms disappear. Third, NoVs are robust and can survive on dry surfaces for several weeks [14]. Finally, NoVs spread by the fecal-oral route, including contaminated water sources, food, and contaminated surfaces [15]. The incubation period is 24–48 h with 12 h the shortest and 72 h the longest reported incubation period. Children present with vomiting and diarrhea while adult patients tend to present with diarrhea alone. The course of the disease is self-limiting and patients usually recover in 2 to 3 days. There are currently no specific antiviral drugs against NoVs and treatment is mainly supportive.

## Epidemiology

### Disease burden

Since acute gastroenteritis (AGE) caused by NoVs is not a notifiable disease in China, the incidence of NoVs cannot be obtained directly from the currently available national statistics. There is also no systematic research into NoV episodes using satisfactory representative sampling. Data from three sources can be used to estimate the NoV specific burden in China: (1) hospital-based sentinel surveillance for diarrhea, (2) retrospective cross-sectional population-based surveys used worldwide for the prevalence of AGE [16], and (3) individual population-based viral AGE surveillance.

A systematic review extracted and synthesized the prevalence of community-based all-cause AGE obtained from the published literatures, and based on NoVs detection rates extrapolated the incidence of NoV AGE [8]. Surprisingly, the estimated NoV incidence for China in 2015 reached 6.0 (95% confidence interval [CI]: 4.7–7.6) per 100 person per year for the entire population with the highest incidence in children less than 5 years of age, 15.6 (95% CI: 9.1–23.2) per 100 children per year [8]. Between 2012 and 2013, an incidence of 8.9 cases (95% CI: 8.2–9.7) per 100 per year for all age groups, with a highest incidence of 20.3 (95% CI: 13.8–27.9), and 14.7 (95% CI: 9.2–20.8) per 100 per year in children aged 0–11 months and 12–23 months respectively, was estimated in Shanghai based on hospital sentinel surveillance [17]. Further evidence comes from abovementioned independent systems. An incidence of 4.7–11.7 per 100 per year in children aged 6–11 months and 4.0–6.6 per 100 per year in children 12–17 months were determined from a population-based surveillance conducted in Zhengding County, Hebei Province and Sanjiang County, Guangxi Zhuang Autonomous Region between 2011 and 2013 [18]. These findings suggest that considerable disease burden is caused by NoV in China. Due to the exclusion of disease episodes which present with vomiting only from nearly all studies, the published data underestimate the actual NoV burden in China.

### Population at risk

Identification of populations at risk for transmission is always crucial for disease control and vaccine development. Children less than 2 years of age are at the highest risk for NoV AGE, followed by young adults and a middle age population (20–50 years), and the elderly ( ⩾ 65 years) based on sentinel surveys for diarrhea in hospitals and the routine community-based cross-section surveys for AGE [8]. The proportion of AGE caused by NoVs found in a nationwide diarrhea survey in hospitals was 13.7% (95% CI: 13.1%–14.3%), 8.7% (95% CI: 7.5%–9.9%), 11.7% (95% CI: 11.1%–12.3%), and 12.3% (95% CI: 11.0%–13.7%) in 6–23 months, 24–59 months, 25–64 years, and elderly ( ⩾ 65 years), respectively [19]. A typical age distribution of rotavirus AGE in children < 5 years was also concluded from a population-based NoV AGE surveillance. The incidence was 4.7–11.7 cases, 4.0–6.6 cases, 2.5–2.9 cases, 1.3–1.9 cases and < 1.0 cases per 100 children per year in children aged 6–11 months, 12–17 months, 18–23 months, 24–29 months and ⩾ 30 months, respectively [18]. Moreover, the severity of illness caused by NoVs is similar to that induced by rotavirus infection [18].

Hospital-based prospective, community-based retrospective and population-based prospective AGE surveillance found that the incidence of NoV AGE decreased with age from 3 to 24 years. The majority (~80%) of NoVs outbreaks reported to the Chinese Public Health Emergency Event Surveillance System (PHEESS) occurred in older children attending kindergartens, primary schools, and secondary schools [20]. The character of the immunity acquired after NoVs infections remains incompletely understood. The current understanding, that NoVs infections confers immunity for 6 months and 2 years comes from human challenge studies, which fails to explain the low incidence of NoV related disease in this population. Children have more opportunities to become exposed to NoVs through direct and indirect contact with other children recorded in the reports of outbreaks, in addition to the sporadic transmission. Thus, a longer persistence can probably be expected than was estimated in the challenge study. Several recent published modeling studies have estimated that immunity against NoVs lasts from 4 to 8 years post-infection [21].

In China, not only young children but also people ⩾ 65 years of age are at an increased risk for NoV AGE. More than 10% NoVs outbreaks were reported from nursing homes and medical institutions for the elderly in the past years in China [22]. A systematic review of 102 NoVs outbreaks from 2003 to 2013 in China, found the highest attack rate 68% in hospitalized elderly patients [23]. A multivariate analysis found the risk of a NoV AGE episode increased with increasing need of nursing support (Odds Ratio = 1.40, 95% CI: 1.03–1.91) [24]. In addition, studies from the United States indicating that, compared to hospitalized children, hospitalized elderly with NoVs infection were more commonly admitted to the intensive care unit (ICU) (36% vs. 7%, $P = 0.02$) [25], and resulted in a 10-fold higher case fatality rate in elderly than in children [26]. China is entering an era of an aging society. The elderly ( $\geqslant$ 65 years) accounted for $>$ 20% of entire population in Shanghai in 2017 [27]. As the consequence of the one-child policy, the traditional home-based care model is no longer realistic in many parts of China, more and more elderly people need to live in nursing homes, and NoV infections will present a serious challenge for public health in China in the future.

AGE remains an unmet concern in military forces. Outbreaks due to NoVs infection have been reported in many countries, for instance, an outbreak involving 450 cases of AGE occurred on a US Naval aircraft carrier in 1997 [28]. AGE outbreaks have been reported in British [29], French [30], Singapore [31] and Chinese [32] military units. NoVs illness is generally mild, characterized by acute vomiting and diarrhea lasting for couple of days, but can be severe when soldiers are exhausted and dehydrated, eventually leading to their diminished operational effectiveness.

### Seasonality

Although the distribution of NoV AGE varies between regions, it is believed to be associated with heavy rainfall, low temperature, and high humidity in mathematical models [33]. Although NoV AGE can occur throughout the year, historically, NoV AGE has been considered a “winter vomiting disease” [34]. In general, more NoV infections are detected during the colder season (October throughout March). This trend was not significant in the provinces in the south of China [8,19]. The incidence of NoVs infection is remarkably low in the Qinghai-Tibet Plateau located in south-west of China [19], probably related to the low humidity in the region. Similar to sporadic NoV AGE, the majority of outbreaks have occurred in the colder season [8,19,20,23,35].

## Genotype diversity

From the nationwide diarrhea survey in hospitals, between 2009 and 2013, 34 031 diarrhea cases were reported from 173 sentinel hospitals, with 3878 NoV infections. Of these, 3484 (89.8%) cases were caused by genogroup II viruses, and only 394 (10.2%) cases infected by genogroup I viruses [19]. Although GI, GII, and GIV NoVs can attack humans, GII is the predominant virus group circulating in China. To understand the genetic diversity and temporal distribution of NoV circulating in China, all available NoVs gene sequences isolated from human host were downloaded from the NCBI GenBank Database by searching the corresponding taxonomy ID of NoVs (Taxonomy ID: 142786) with a keyword “China.” As of May 24, 2016, in total, 3134 NoV sequences detected in China were analyzed. Only GI and GII viruses were detected, which accounted for 13.1% (411) and 86.9% (2723), respectively. Overall, 12 GI genotypes and 18 GII genotypes were identified. The most common genotype was GII.4 (1571; 50.1%), followed by GII.17 (343; 10.9%) and GII.3 (240; 7.7%), other common genotypes included GI.2 (108; 3.4%), GII.12 (107; 3.4%), GII.e (104; 3.3%), GII.6 (99; 3.2%), GI.3 (70; 2.2%), GI.5 (65; 2.1%), GI.1 (51; 1.6%), GI.4 (44; 1.4%), GII.13 (39; 1.2%), and GII.21 (37; 1.2%) (Table 1) [36].

Due to the preferential reporting of outbreaks and emerging strains, the distribution of genotype summarized from those GenBank’s sequences might not be representative of the real genotype distribution in the population. For instance, GII.17 had been an emergent strain causing a number of outbreaks in China in 2014 and 2015. It spread rapidly since 2013, from an initial 4.0% to 79.6% in 2015, and eventually accounted for 10.9% of GenBank sequences. To eliminate the potential bias due to over-reporting of outbreak strains, a meta-analysis was conducted which included reports published before January 2017, which found the most common genotype was GII.4 (49.7%), followed by GII.3 (9.5%) and GII.17 (8.4%), other common genotypes included GII.12 (2.4%), GI.2 (2.3%), GI.4 (1.8%), GI.3 (1.7%), GII.13 (1.7%), GII.6 (1.5%), and GI.5 (1.4%) (Table 1) [8].

Population-based surveillance in children $<$ 5-year conducted in Zhengding and Sanjiang County between 2011 and 2013 found a similar genotype distribution. In Zhengding, the predominant genotype were GII.3 (32.5%) and GII.4 (30.0%), followed by GII.2 (7.5%) and GII.6 (2.5%); in Sanjiang, majority infection were caused by GII.6 (39.3%) and GII.4 (37.7%), followed by GII.7 (4.9%) and GII.17 (3.3%) (Table 1) [18].

NoV genotypes vary with time, region, and age group but the epidemic genotype did not change frequently in China. Since the late 1990s, a single genotype (GII.4) of NoVs has been dominant. Six variants of GII.4 have emerged during the period. The most prevalent variants were Den Haag_2006b in 2006–2011, New Orleans_2009 in 2009–2011, and Sydney_2012 in 2012–2015; the other

**Table 1** Genotypes distribution of norovirus by study design in China

| Genotype | Sources of data and study design | | | |
|---|---|---|---|---|
| | GenBank (hospital-based and outbreaks surveys) (%) | Meta-analysis (hospital-based surveys) (%) | Zhengding (population-based surveillance) (%) | Sanjiang (population-based surveillance) (%) |
| GI.1 | 1.6 | 0.6 | – | – |
| GI.2 | 3.4 | 2.3 | – | – |
| GI.3 | 2.2 | 1.7 | – | – |
| GI.4 | 1.4 | 1.8 | 0.6 | – |
| GI.5 | 2.1 | 1.4 | – | – |
| GI.6 | 0.7 | 1.3 | – | – |
| GI.7 | 0.1 | 1.3 | – | – |
| GI.8 | 0.8 | 0.8 | – | – |
| GI.9 | 0.2 | – | – | 1.6 |
| GII.1 | 0.1 | 2.8 | – | – |
| GII.2 | 0.9 | 0.8 | 7.5 | – |
| GII.3 | 7.7 | 9.5 | 32.5 | – |
| GII.4 | 50.1 | 49.7 | 30.0 | 37.7 |
| GII.5 | 0.1 | 2.4 | – | – |
| GII.6 | 3.2 | 1.5 | 2.5 | 39.3 |
| GII.7 | 0.7 | 0.4 | 0.6 | 4.9 |
| GII.8 | 0.03 | 0.5 | – | – |
| GII.10 | – | 0.8 | – | – |
| GII.11 | – | 1.7 | – | – |
| GII.12 | 3.4 | 2.4 | – | – |
| GII.13 | 1.2 | 1.7 | – | – |
| GII.14 | 0.2 | 0.4 | – | – |
| GII.15 | 0.1 | 0.6 | – | – |
| GII.16 | 0.1 | 0.5 | – | – |
| GII.17 | 10.9 | 8.4 | – | 3.3 |
| GII.18 | – | 0.6 | – | – |
| GII.21 | 1.2 | 0.4 | – | – |
| GII.22 | 0.03 | 0.8 | – | – |
| Others | 7.46 | 2.8 | 26.3 | 13.1 |

three minor variants were Asia_2003 in 2004–2005, Hunter_2004 in 2004–2005, and Apeldoorn_2007 in 2009, respectively [37]. New GII.4 strains emerge almost every 2–4 years, and associated with an increased NoVs activity. It is noteworthy that, in addition to GII.4, a novel variant of GII.17 and a recombinant variant of GII.2 (GII.P16/GII.2) had a wide distribution between 2014 and 2015 and 2016 – 2017, respectively [38–41].

## Contaminated food and other environmental sources for NoV infections

NoVs illness is commonly contracted through contaminated food, water and person-to-person transmission. NoVs have been detected in shellfish [42,43], lettuce, strawberries [44], river water [45], sewage [46], and environmental samples [47] in the past years. Despite concerted efforts to improve food safety, the food- and water-borne outbreaks continue, since some products, including salads and shellfish are typically eaten raw or only lightly cooked.

China possess 45 coastal fishing regions between Liaoning Province in the North to Hainan Province in the South. To get a better understanding of the contamination of economically important shellfish with human enteric viruses, a study was conducted which investigated samples from the abovementioned 45 regions. Contaminated shellfish were detected in all regions, with an average detection rate of 14.8% (24/162). No significant difference in detection rate was observed between the coastal fishing regions. Of 6 shellfish groups (clams, oysters, mussels, razor clams, blood clams, and scallops), oysters had the highest detection rate (35%) of NoVs, followed by blood clams (22%) [43]. These results suggest that uncooked seafood is a potential public health hazard in China.

Studies have established a close correlation between seafood, aquaculture water, sewage, river water, and AGE. A study conducted in Guangdong Province explored the effect of seasonality of the detection rates. During winter and spring more NoVs were detected in aquaculture water (20.0%), seafood (50.0%) and human AGE patients (20.7%), compared to summer and autumn (6.2%, 10.9% and 17.6%) (Fig. 1), though the seasonality in patients did not reach statistical significance [48]. Phylogenetic analyses suggests that homologous NoV genotypes circulate between human populations, seafood and aquaculture water [48]. This suggestion is supported by studies conducted in Guangdong Province. For instance, 19.7% (36/183) of water specimens collected over an 18 months period from the Zhu River (the river passing through Guangzhou City) were NoVs positive. Homologous sequences were identified from river water, seafood, and AGE patients [45]. It has been suggested that NoVs shed from patients re-enter the river, float to coastal fishing areas downstream, where seafood is subsequently contaminated.

As for person-to-person transmission, NoVs could be detected on soap boxes (63.6%, 7/11), handle of mops (50.0%, 5/10), cups (44.4%, 4/9), toilet-doors (40.0%, 2/5), toys (22.2%, 2/9), water taps (16.7%, 1/6), and tableware (11.1%, 1/9) in two kindergartens during the outbreak [47]. Moreover, NoVs genomes were detected in air samples taken in 3 distinct locations on patient wards: (1) inside the room of patients with AGE symptoms ( $<$ 24 h), (2) in the hallways or the common room outside of the rooms of patients with symptoms, and (3) at the nurses' station, where concentrations ranged from $1.35 \times 10^1$ to $2.35 \times 10^3$ genomes/m$^3$ in 47% of air samples [49].

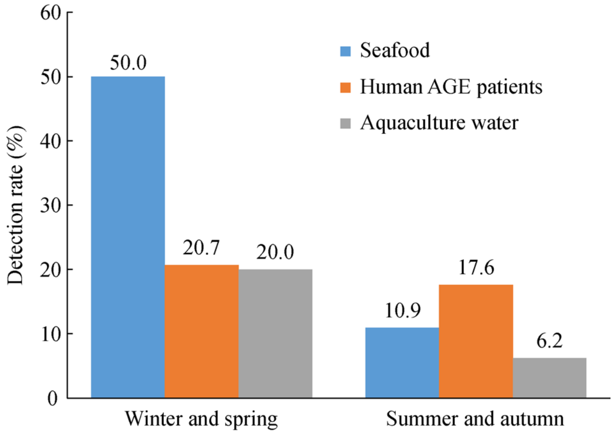

**Fig. 1** The detection rates of norovirus from different sources by seasonality.

## Summary

Similar to the occurrence and epidemiological characteristics of NoVs worldwide [2], NoVs also cause a considerable disease burden in China, especially in children $<$ 2 years and elderly $\geqslant$ 65 years. Public awareness of NoV infections is increasing, due to frequent reports of outbreaks. Since NoVs are widespread in food supplies and environment in China, these potential vehicles might make NoV transmission ubiquitous. Given their highly infectious nature, propensity for causing outbreaks, primary environmental reservoir, and tolerance to physical and chemical substances, vaccination appears to be the most appropriate strategy to control NoV infections. Recently, two vaccine candidates, both based on the expression of NoV virus-like particles (VLPs), being composed of GI.1, GII.4 and GI.1, GII.3, GII.4, GII.17, respectively, have been approved for human trials in China. However, NoVs are antigenically diverse, and it is currently not known whether the cross-protection between genotypes exists. In addition, there is currently no well-established immunological surrogate to assess the potential efficacy of vaccine candidates. These challenges for vaccine development need to be addressed urgently to help control NoV infections.

## Acknowledgements

This work was supported by grants from National Science and Technology Project of Major New Drug Discovery (No. 2018ZX09739002-006).

## Compliance with ethics guidelines

Honglu Zhou, Songmei Wang, Lorenz von Seidlein, and Xuanyi Wang declare that they have no conflict of interest. This manuscript is a review article and does not involve a research protocol requiring approval by a relevant institutional review board or ethics committee.

**Open Access** This article is licensed under a Creative Commons Attribution 4.0 International License, which permits use, sharing, adaptation, distribution and reproduction in any medium or format, as long as you give appropriate credit to the original author(s) and the source, provide a link to the Creative Commons license, and indicate if changes were made.

The images or other third party material in this article are included in the article's Creative Commons license, unless indicated otherwise in a credit line to the material. If material is not included in the article's Creative Commons license and your intended use is not permitted by statutory regulation or exceeds the permitted use, you will need to obtain permission directly from the copyright holder.

To view a copy of this license, visit http://creativecommons.org/licenses/by/4.0/.
